# Supplementary material for: Predicting cardiovascular disease risk using photoplethysmography and deep learning
Source: PLOS Glob Public Health. 2024 Jun 4;4(6):e0003204. doi: 10.1371/journal.pgph.0003204 (PMC11149850; doi:10.1371/journal.pgph.0003204)

**S1 Fig. Overview of our deep learning-based risk prediction model, DLS.** Blue: models; yellow: inputs; red: intermediate data representations (embeddings) obtained from the deep learning-based PPG feature extractor.


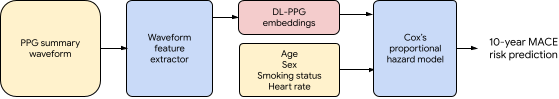

Supplement: S1 Fig — Blue: models; yellow: inputs; red: intermediate data representations (embeddings) obtained from the deep learning-based PPG feature extractor. (DOCX) [file pgph.0003204.s001.docx]
