# Supplementary material for: Predicting cardiovascular disease risk using photoplethysmography and deep learning
Source: PLOS Glob Public Health. 2024 Jun 4;4(6):e0003204. doi: 10.1371/journal.pgph.0003204 (PMC11149850; doi:10.1371/journal.pgph.0003204)

**S2 Fig. Geographical location information of sites visualized by longitude and latitude for dataset splits.**

#
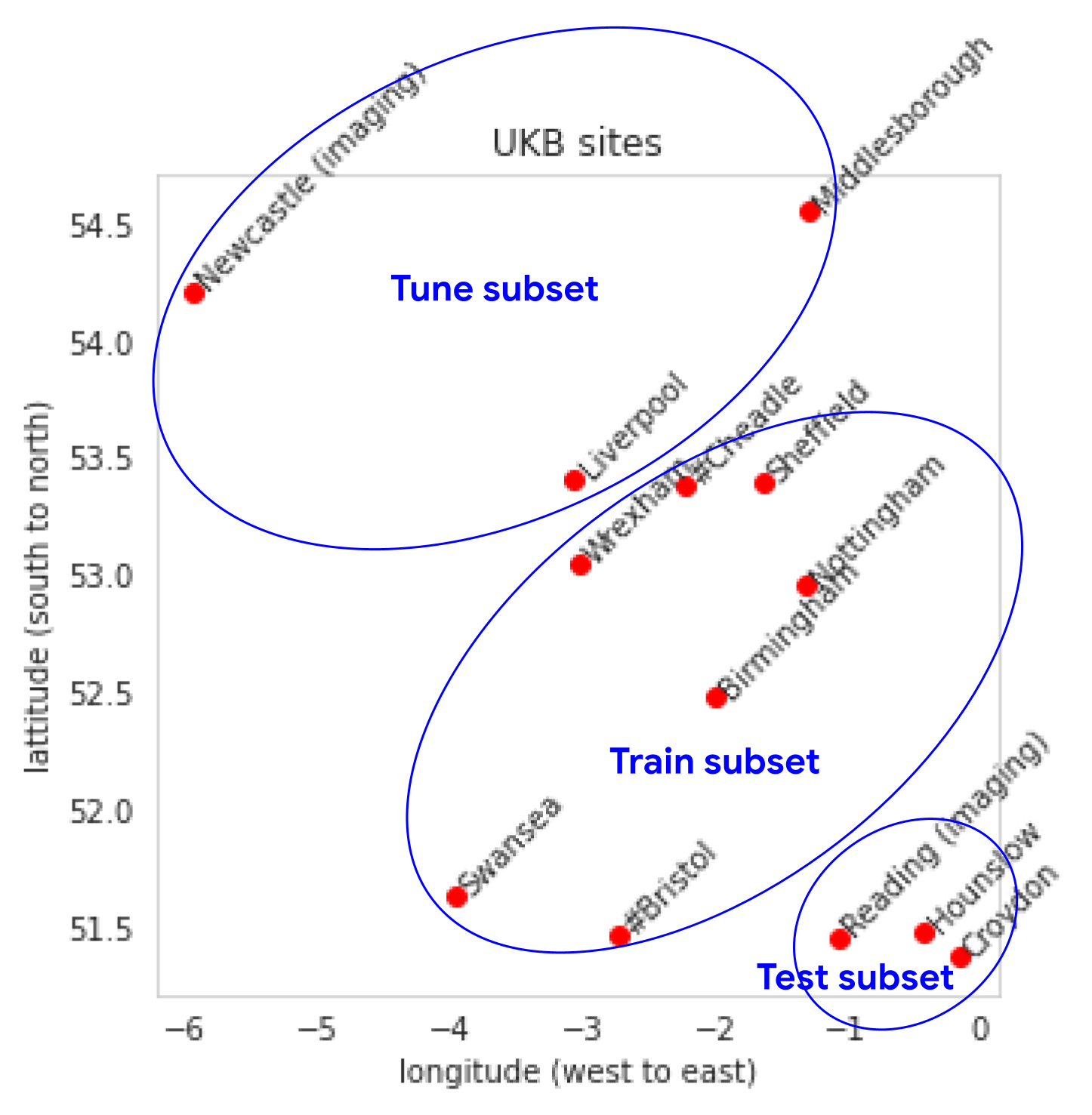

Supplement: S2 Fig — (DOCX) [file pgph.0003204.s002.docx]
