# Supplementary material for: Predicting cardiovascular disease risk using photoplethysmography and deep learning
Source: PLOS Glob Public Health. 2024 Jun 4;4(6):e0003204. doi: 10.1371/journal.pgph.0003204 (PMC11149850; doi:10.1371/journal.pgph.0003204)

**S3 Fig. Kaplan-Meier estimation of DLS with different operating points.** We compared the survival estimation between the high and low risk groups, which were defined by the risk threshold at 10% suggested by the Globorisk study [[1]](https://paperpile.com/c/hCP1h7/Ctml). For example, a case with prediction value higher than 0.1 will be high risk, else low risk. The p-values were calculated by the log-rank test.


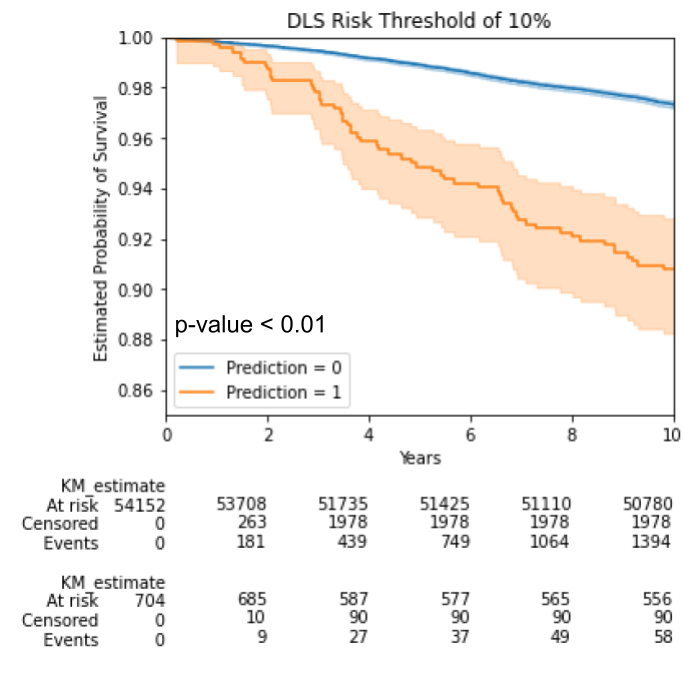

Supplement: S3 Fig — We compared the survival estimation between the high and low risk groups, which were defined by the risk threshold at 10% suggested by the Globorisk study [1]. For example, a case with prediction value higher than 0.1 will be high risk, else low risk. The p-values were calculated by the log-rank test. (DOCX) [file pgph.0003204.s003.docx]
