# Supplementary material for: Predicting cardiovascular disease risk using photoplethysmography and deep learning
Source: PLOS Glob Public Health. 2024 Jun 4;4(6):e0003204. doi: 10.1371/journal.pgph.0003204 (PMC11149850; doi:10.1371/journal.pgph.0003204)

**S4 Fig. Calibration plots for all subgroups.** The calibration slope values indicate the coefficient of a linear regression where the dependent variable was the fraction of positives (predicted risk) and the independent variable was the mean prediction. We used ten bins to discretize the prediction interval and chose deciles of predicted risk to define the widths of the bins. For the elevated HbA1c subgroup, we used quintiles to ensure sufficient events. All models (office-based refit-WHO, DLS) are calibrated better in smoking, older, male, non A1c elevated, and non-hypertensive subgroups.


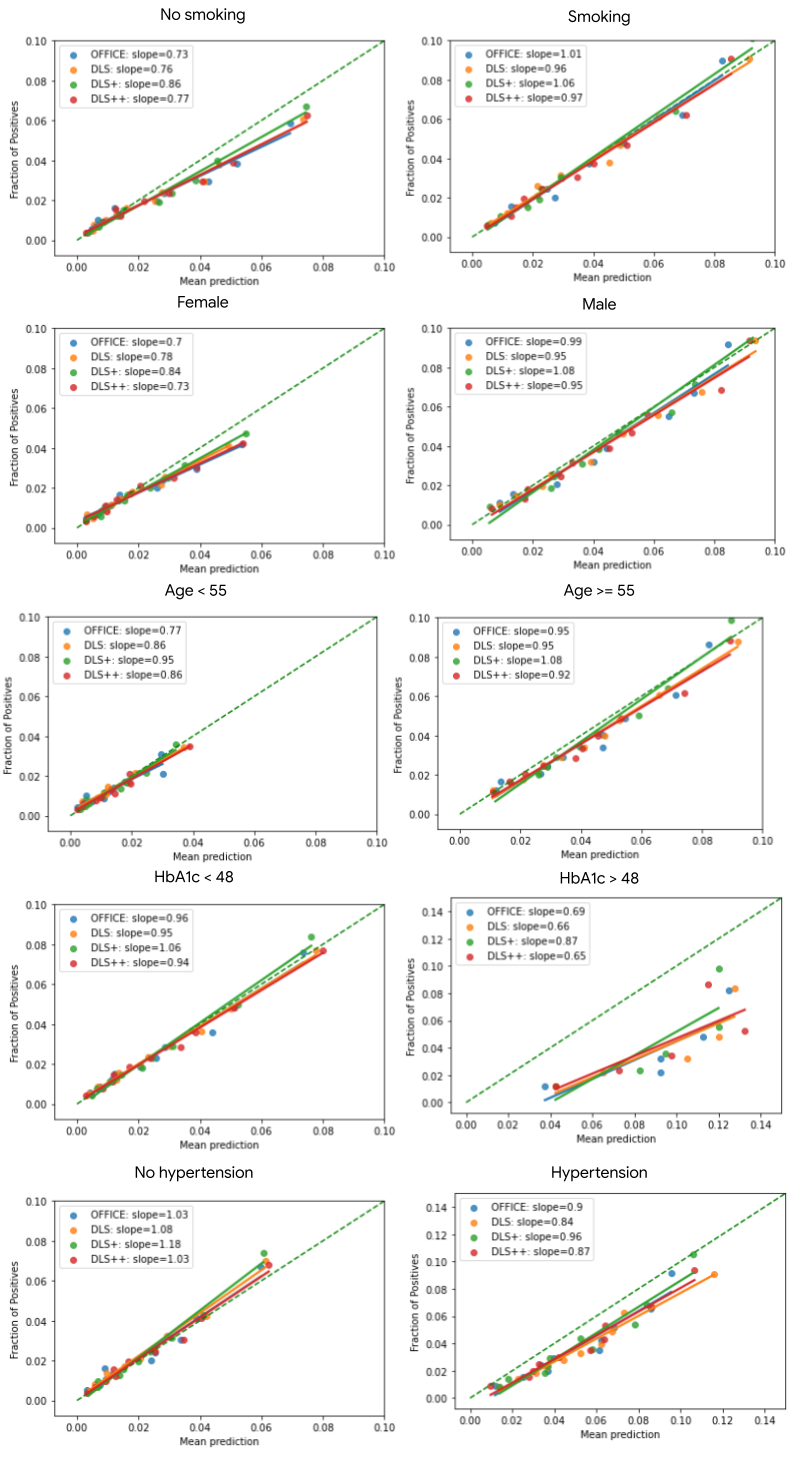

Supplement: S4 Fig — The calibration slope values indicate the coefficient of a linear regression where the dependent variable was the fraction of positives (predicted risk) and the independent variable was the mean prediction. We used ten bins to discretize the prediction interval and chose deciles of predicted risk to define the widths of the bins. For the elevated HbA1c subgroup, we used quintiles to ensure sufficient events. All models (office-based refit-WHO, DLS) are calibrated better in smoking, older, male, non A1c elevated, and non-hypertensive subgroups. (DOCX) [file pgph.0003204.s004.docx]
