# Supplementary material for: Predicting cardiovascular disease risk using photoplethysmography and deep learning
Source: PLOS Glob Public Health. 2024 Jun 4;4(6):e0003204. doi: 10.1371/journal.pgph.0003204 (PMC11149850; doi:10.1371/journal.pgph.0003204)

**S5 Fig. Prevalence of major adverse cardiovascular event (MACE) in individuals according to model-predicted risk percentiles.** For each of four risk models, the prevalence of MACE was computed in the individuals scoring in the highest 20, 10, and 5% risk according to the model. Error bars computed via 100 bootstrap iterations. The dashed gray line shows MACE prevalence in the entire sample. Metadata+, model containing age, sex, smoking status, and BMI. DLS+, model containing age, sex, smoking status, BMI, and PPG. Metadata+ + polygenic risk score (PRS), model containing age, sex, smoking status, BMI, and polygenic risk score. DLS+ + PRS, model containing age, sex, smoking status, BMI, PPG, and PRS.


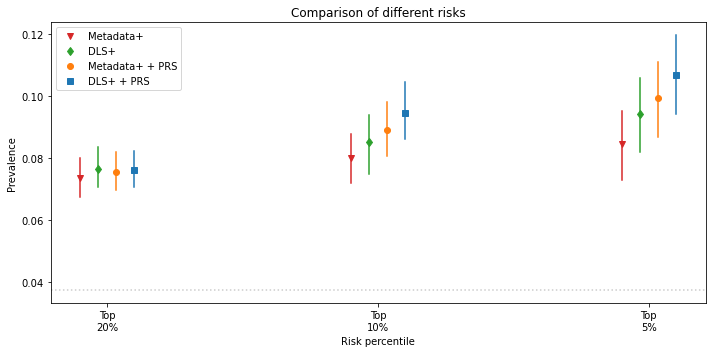

Supplement: S5 Fig — For each of four risk models, the prevalence of MACE was computed in the individuals scoring in the highest 20, 10, and 5% risk according to the model. Error bars computed via 100 bootstrap iterations. The dashed gray line shows MACE prevalence in the entire sample. Metadata+, model containing age, sex, smoking status, and BMI. DLS+, model containing age, sex, smoking status, BMI, and PPG. Metadata+ + polygenic risk score (PRS), model containing age, sex, smoking status, BMI, and polygenic risk score. DLS+ + PRS, model containing age, sex, smoking status, BMI, PPG, and PRS. (DOCX) [file pgph.0003204.s005.docx]
