# Supplementary material for: Predicting cardiovascular disease risk using photoplethysmography and deep learning
Source: PLOS Glob Public Health. 2024 Jun 4;4(6):e0003204. doi: 10.1371/journal.pgph.0003204 (PMC11149850; doi:10.1371/journal.pgph.0003204)
Supplement: S1 Text — (DOCX) [file pgph.0003204.s006.docx]

## S1 Text. Supporting methods

### Details of UK Biobank photoplethysmography data

The photoplethysmography (PPG) waveforms in the UKB (Data field 4205) were acquired using the PulseTrace PCA2 device (CareFusion, USA). The device collected and averaged a minimum of six heart beats per user with a pulse interval close to the average pulse interval. It has been shown that morphological properties of a single representative PPG waveform are related to CV aging and CVD risk, such as augmentation index [[4–6]](https://paperpile.com/c/hCP1h7/XoG0+y4VR+dHyS). We preprocessed PPG waveforms by re-scaling to [0, 1].

### Details of model training

During model training, we adopted a multitask learning framework with multiple proxy prediction tasks, such as age, sex, BMI, blood pressure, laboratory data predictions (S9 Table).

We also used a custom data augmentation inspired by Brownian motion which we termed Brownian tape speed augmentation. We developed the Brownian tape speed augmentation technique, inspired by Brownian motion, to improve the generalizability of the model. The method simulates playing back the signal on a tape while the tape’s playback speed is varying according to Brownian motion. Specifically, the playback speed at each time step is drawn from a normal distribution. The method has a single hyperparameter, which we call the magnitude, that is used to define the standard deviation of this normal distribution. For each sequence (i.e., PPG signal), the magnitude is divided by the sequence length to set the standard deviation of the normal distribution for that sequence. This division ensures that regardless of the length of the sequence, the overall amount of transformation is similar.

We then calculate a running sum of this array of normal distribution samples, and add 1 everywhere in order to simulate a random walk of tape speed starting at 1. The array now represents the tape speed. We then calculate another running sum, and now the array represents displacement. We use this displacement as a flow field which is then applied to transform the input using the tfa_image.dense_image_warp function in tensorflow.

Training setup for the PPG feature extractor is listed in S2 Table.

### Photoplethysmography morphology-based features

In the metadata + PPG morphology model, we used the engineered features available in the UK Biobank for PPG-based arterial stiffness evaluation (S4 Table). The features are pulse wave reflection index (RI), peak to peak time, pulse wave peak position, pulse wave notch position, pulse wave shoulder position, the presence/absence of dicrotic notch, and arterial stiffness index derived from the peak to peak time and the height of the participant.

### Polygenic risk model creation

Individuals of European genetic ancestry who did not have PPG data were split into genome-wide association study (GWAS) (n=208k), train (n=40k), and tune (n=40k) sets. We performed GWAS on the GWAS dataset using BOLT-LMM v2.3.6 [[7]](https://paperpile.com/c/hCP1h7/RONs) and adjusting for age, sex, genotyping array, smoking status, and the top 15 genetic principal components for the following 24 cardiovascular disease-related phenotypes: angina, myocardial infarction, coronary artery disease, heart failure, stroke, cardiovascular death, hypertension, atrial fibrillation, rheumatic heart disease, rheumatoid arthritis, chronic renal failure, diabetes, systolic blood pressure, diastolic blood pressure, low-density lipoprotein (LDL) cholesterol, high-density lipoprotein (HDL) cholesterol, total cholesterol, triglycerides, hemoglobin A1C, glucose, boday mass index (BMI), and three definitions of major adverse cardiovascular event (MACE) as the logical OR of myocardial infarction, cardiovascular death, and stroke; the logical OR of myocardial infarction, cardiovascular death, and heart failure; and the logical OR of myocardial infarction, cardiovascular death, stroke, heart failure, angina, and coronary artery disease (MACE-lenient). For each phenotype, a polygenic risk score (PRS) was generated by BOLT-LMM using the --predBetasFile option. Additionally, we ran PolyFun [[8]](https://paperpile.com/c/hCP1h7/teCA) to create functionally-informed fine-mapping PRS. We trained a multilayer perceptron to predict the MACE-lenient phenotype from the 48 PRSs in the train set and selected hyperparameters based on performance in the tune set. We then applied the model to all individuals with PPG data and used the resulting model prediction as the MACE PRS.
