# Supplementary material for: Predicting cardiovascular disease risk using photoplethysmography and deep learning
Source: PLOS Glob Public Health. 2024 Jun 4;4(6):e0003204. doi: 10.1371/journal.pgph.0003204 (PMC11149850; doi:10.1371/journal.pgph.0003204)
Supplement: S2 Text — (DOCX) [file pgph.0003204.s007.docx]

## S2 Text. Supporting results

### Analysis for the fixed model operating point at 10% risk

At the threshold of 10% risk suggested by the Globorisk study, the sensitivity, specificity, and NRI of DLS was 4.0% (3.0, 5.1), 98.9% (98.8, 99.0), 0.6% (-0.6, 1.7), respectively. Meanwhile, the sensitivity and specificity of the office-based refit-WHO was 3.0% (2.2, 4.0), 99.1% (99.0, 99.2), respectively. We found that without any medical device-dependent measurement, DLS is non-inferior to the office-based refit-WHO score given the risk threshold of 10% suggested by Globorisk study. The full evaluation of all models is listed in the S5 Table.

### Models without smoking status

We further examined Cox's models without using smoking status as a feature (the office-based refit-WHO score without smoking status, DLS without smoking status). We found that removing smoking status from the predictor set did not reduce the DLS performance and non-inferiority relative to the office-based refit-WHO was maintained (S10 Table). However, the calibration was worse (slope of 0.968).

### Applying additional features helps improve the cardiovascular disease risk prediction

In S6 Table, we demonstrated that adding BMI and SBP on top of the DLS model helps improve the model performance on ten-year MACE risk prediction. We also showed that the lab-based refit-WHO model outperformed the office-based model on C-statistic and specificity matching the sensitivity. Meanwhile, we developed a Full model that included most risk factors used in QRISK and/or ASCVD [[9,10]](https://paperpile.com/c/hCP1h7/pP1M+pHwL)—age, ethnicity, deprivation (IMD score), sex, smoking status, BMI, SBP, glucose level, total cholesterol, HDL, medication for hypertension, past medical history of angina, chronic kidney disease, diabetes, erectile dysfunction, mental illness, rheumatoid arthritis, and systemic lupus erythematosus. The Full model was compared with the office-based reference on a smaller cohort subset due to missing data. The Full model yielded the C-statistic of 73.5% (72.3, 74.8), while the DLS got 71.3% (70.0, 72.6), and the office-based model got 71.2% (70.0, 72.5) (superiority test p<0.01 for both) on the same UKB subset. Regarding the specificity matching the sensitivity of 55.2%, the Full model was 76.5% (76.1, 76.9), while the DLS was 74.3% (72.2, 76.6), and the office-based model was 73.7% (73.3, 74.1) (both p<0.01). While matching the specificity of 63.7%, the sensitivity was 71.1% (68.5, 73.6) versus 68.2% (65.5, 70.8) for DLS and 68.8% (66.2, 71.4) for the office-based model (both p<0.01). We concluded that the Full model showed a better MACE prediction on the UKB subset with the variables available for analysis.

In S5 Fig, we investigated the extent to which individuals predicted to be at high risk by a model are enriched for MACE prevalence. As expected given the observed improvement in C-statistic, the DLS+ model shows superior performance to the Metadata+ model that contains only age, sex, smoking status, and BMI when examining MACE prevalence in the top 5% and 10% of predicted risk. We observed a similar, and slightly more pronounced, improvement from a model that includes a PRS component in addition to the Metadata+ features at the same 5% and 10% most extreme risk percentiles (2.39- and 2.67-fold enrichment over total sample prevalence, respectively, compared to 2.14- and 2.26-fold enrichment for Metadata+). Interestingly, the contributions of PPG and genetic risk appear complementary, as a model that includes Metadata+, PPG, and PRS was most enriched for MACE prevalence (2.53- and 2.87-fold enrichment, respectively).

While both the Full model and the model including polygenic risk show improved MACE prediction performance, we note that each requires more variables that may not be available in low-resource settings, which may limit the use of such lab-based approaches like QRISK, ASCVD, and PRS.
