# Supplementary material for: Predicting cardiovascular disease risk using photoplethysmography and deep learning
Source: PLOS Glob Public Health. 2024 Jun 4;4(6):e0003204. doi: 10.1371/journal.pgph.0003204 (PMC11149850; doi:10.1371/journal.pgph.0003204)
Supplement: S1 Table — (DOCX) [file pgph.0003204.s008.docx]

**S1 Table. Geographical location information of sites for split division.** The visualized version is in S2 Fig.

| **Subset** | **Site Name** | **Region** | **Latitude** | **Longitude** |
| --- | --- | --- | --- | --- |
| Train  (9 sites,  97,970 participants) | Swansea | Center | 51.6255141 | -3.9655064 |
|  | Bristol | Center | 51.4684055 | -2.7308013 |
|  | Bristol (imaging) | Center | 51.4684055 | -2.7308013 |
|  | Birmingham | Center | 52.4773549 | -2.0037151 |
|  | Nottingham | Center | 52.9539402 | -1.3101453 |
|  | Sheffield | Center | 53.3956347 | -1.6395396 |
|  | Cheadle (imaging) | Center | 53.3877966 | -2.2245145 |
|  | Cheadle (revisit) | Center | 53.3877966 | -2.2245145 |
|  | Wrexham | Center | 53.0510806 | -3.0231874 |
| Tune  (3 sites, 43,539 participants) | Newcastle (imaging) | North-West | 54.2129983 | -5.9309897 |
|  | Middlesborough | North-West | 54.5545144 | -1.2930867 |
|  | Liverpool | North-West | 53.4120954 | -3.0561422 |
| Test  (3 sites,  54,856 participants) | Croydon | South-East | 51.3678072 | -0.1522794 |
|  | Hounslow | South-East | 51.4759853 | -0.4163816 |
|  | Reading (imaging) | South-East | 51.4514358 | -1.066087 |
