# Supplementary material for: Predicting cardiovascular disease risk using photoplethysmography and deep learning
Source: PLOS Glob Public Health. 2024 Jun 4;4(6):e0003204. doi: 10.1371/journal.pgph.0003204 (PMC11149850; doi:10.1371/journal.pgph.0003204)
Supplement: S2 Table — (DOCX) [file pgph.0003204.s009.docx]

**S2 Table. Training setup for the photoplethysmography (PPG) feature extractor.**

| **Hyperparameter** | **DLS** | **DLS+** | **DLS++** |
| --- | --- | --- | --- |
| Neural network architecture | ResNet-18 | | |
| Dropout rate | 0.0 | | |
| Epochs | 80 | | |
| Optimizer | AdamW [[11]](https://paperpile.com/c/hCP1h7/U1gE) | | |
| Learning rate | 0.0001 with cosine 1-epoch warmup | 0.0003 with cosine 1-epoch warmup | 0.0001 with cosine 1-epoch warmup |
| Weight decay | 0.000003 | 0.0001 | 0.000003 |
| Augmentation | Brownian tape speed with magnitude of 2 and randomly applied 50% (1/2) of the time | Brownian tape speed with magnitude of 0.1 and randomly applied 50% (1/2) of the time | Brownian tape speed with magnitude of 2 and randomly applied 50% (1/2) of the time |
| Ridge penalization parameter for the Cox model | 0.00003 | 0.00003 | 0.00003 |
