# Supplementary material for: Predicting cardiovascular disease risk using photoplethysmography and deep learning
Source: PLOS Glob Public Health. 2024 Jun 4;4(6):e0003204. doi: 10.1371/journal.pgph.0003204 (PMC11149850; doi:10.1371/journal.pgph.0003204)
Supplement: S3 Table — We compared all methods with the office-based refit-WHO model. The evaluations of DLS models and additional reference methods are in the main content, S5, S6 and S10 Tables. For the supporting reference methods, the results are listed in the Supporting Tables. *Lab-based refit-WHO and metadata + PPG morphology models are compared with a subset of the whole cohort. **The full model used most QRISK features. The detail of the feature set is described in the Supporting Methods. (DOCX) [file pgph.0003204.s010.docx]

**S3 Table. Features used in different models for comparison.** We compared all methods with the office-based refit-WHO model. The evaluations of DLS models and additional reference methods are in the main content, S5, S6, S10 Tables. For the supporting reference methods, the results are listed in the Supporting Tables. *Lab-based refit-WHO and metadata + PPG morphology models are compared with a subset of the whole cohort. **The full model used most QRISK features. The detail of the feature set is described in the Supporting Methods.

| **Method** | **Age** | **Sex** | **Smoking status** | **BMI** | **SBP** | **Labs** | **Medical history / family history / medications** | **PPG (engineered)** | **PPG (deep learning-based)** |
| --- | --- | --- | --- | --- | --- | --- | --- | --- | --- |
| DLS | x | x | x |  |  |  |  |  | x |
| DLS+ | x | x | x | x |  |  |  |  | x |
| DLS++ | x | x | x | x | x |  |  |  | x |
| Main reference method | | | | | | | | | |
| Office-based refit-WHO | x | x | x | x | x |  |  |  |  |
| Additional reference methods | | | | | | | | | |
| Metadata | x | x | x |  |  |  |  |  |  |
| Lab-based refit-WHO* | x | x | x |  | x | x |  |  |  |
| Metadata + PPG morphology* | x | x | x |  |  |  |  | x |  |
| Supporting reference methods | | | | | | | | | |
| Smoking status-only |  |  | x |  |  |  |  |  |  |
| Office without smoking status | x | x |  | x | x |  |  |  |  |
| DLS without smoking status | x | x |  |  |  |  |  |  | x |
| Full** | x | x | x | x | x | x | x |  |  |
