# Supplementary material for: Predicting cardiovascular disease risk using photoplethysmography and deep learning
Source: PLOS Glob Public Health. 2024 Jun 4;4(6):e0003204. doi: 10.1371/journal.pgph.0003204 (PMC11149850; doi:10.1371/journal.pgph.0003204)
Supplement: S4 Table — (DOCX) [file pgph.0003204.s011.docx]

**S4** **Table. UK Biobank variables used in the study.**

| **Variable** | **UK Biobank data field** | **Notes** |
| --- | --- | --- |
| UKB site | 54 | For data split |
| Visit date | 53 | To identify the age at visit |
| Age | 21003 |  |
| Sex | 31 |  |
| Smoking status | 20116 |  |
| Ethnicity | 21000 | For Full model |
| Deprivation | 26410, 26426, 26427 | For Full model |
| BMI | 21001 |  |
| SBP | 4080 |  |
| Glucose | 30740 | For lab-based score |
| Total cholesterol | 30690 | For lab-based score |
| HbA1c | 30750 | For subgroup analysis |
| HDL | 30760 | For Full model |
| LDL | 30780 | For Full model |
| PPG waveform | 4205 | For all DLS scores |
| PPG pulse rate | 4194 | For all DLS scores and the score using engineered PPG morphology |
| PPG reflection index | 4195 | For the score using engineered PPG morphology |
| PPG peak-to-peak time | 4196 | For the score using engineered PPG morphology |
| PPG peak position | 4198 | For the score using engineered PPG morphology |
| PPG notch position | 4199 | For the score using engineered PPG morphology |
| PPG shoulder position | 4200 | For the score using engineered PPG morphology |
| PPG notch absent | 4204 | For the score using engineered PPG morphology |
| PPG ASI | 21021 | For the score using engineered PPG morphology |
| Hypertension | 6150 (self-reported), 131286 (ICD), 131288 (ICD), 131290 (ICD), 131292 (ICD) | For subgroup analysis |
| Myocardial infarction | 3894 (age at diagnosis), 6150 (self-reported), 42000 (self-reported), 131298 (ICD), 131300 (ICD), 131302 (ICD) | MACE outcome |
| Stroke | 4056 (age at diagnosis), 6150 (self-reported), 131368 (ICD), 131366 (ICD), 42006 (self-reported/EHR), 42008 (self-reported/EHR) | MACE outcome |
| Cardiovascular-related death | 40000 (ICD), 40001 (ICD), 40010 (text: cause of death) | MACE outcome, we considered all cause of death related to coronary artery diseases, myocardial infarction, stroke, hypertensive problems (including ICH, SAH, AAA, etc.), and other heart-related problems |
| Medication | 20003 (self-reported for all medications), 6177 (for statin, hypertension medication, insulin) | For Full model |
| Medical conditions | 20002 (self-reported) | For the Full model, including all self-reported medical history information, e.g. angina, heart failure, hyperlipidemia, erectile dysfunction, mental illness, migraine, chronic kidney disease, rheumatoid arthritis, SLE, etc. |
