# Supplementary material for: Predicting cardiovascular disease risk using photoplethysmography and deep learning
Source: PLOS Glob Public Health. 2024 Jun 4;4(6):e0003204. doi: 10.1371/journal.pgph.0003204 (PMC11149850; doi:10.1371/journal.pgph.0003204)
Supplement: S5 Table — The sensitivity, specificity, and net reclassification improvement (NRI) were calculated at the 10% risk threshold suggested by the Globorisk study for the British population [1]. CIs of sensitivity and specificity were obtained from the Clopper-Pearson exact method, and the p-values were calculated by the permutation test with a prespecified margin of 2.5% and alpha of 0.05. The 95% CIs of NRI were computed by bootstrapping. (DOCX) [file pgph.0003204.s012.docx]

**S5 Table. Model performance comparison of 10-year major adverse cardiovascular event (MACE) risk prediction between DLS versus other methods at the 10% risk threshold.** The sensitivity, specificity, and net reclassification improvement (NRI) were calculated at the 10% risk threshold suggested by the Globorisk study for the British population [[1]](https://paperpile.com/c/hCP1h7/Ctml). CIs of sensitivity and specificity were obtained from the Clopper-Pearson exact method, and the p-values were calculated by the permutation test with a prespecified margin of 2.5% and alpha of 0.05. The 95% CIs of NRI were computed by bootstrapping.

|  | **Sensitivity@risk threshold=0.1** | | | | **Specificity@risk threshold=0.1** | | | |  | | |
| --- | --- | --- | --- | --- | --- | --- | --- | --- | --- | --- | --- |
| **Model** | **Mean (%)** | **Delta (%)** | **Non-inferiority**  **p-value** | **Superiority p-value** | **Mean (%)** | **Delta (%)** | **Non-inferiority**  **p-value** | **Superiority p-value** | **NRI (%)** | **NRI (event) (%)** | **NRI (non-event) (%)** |
| Office-based refit-WHO | 3.0 (2.2, 4.0) | reference | | | 99.1 (99.0, 99.2) | reference | | | | | |
| DLS | 4.0 (3.0, 5.1) | 1.0 (-0.3, 2.1) | <0.01 | 0.082 | 98.9 (98.8, 99.0) | -0.2 (-0.3, -0.1) | <0.01 | 0.999 | 0.6 (-0.6, 1.7) | 0.8 (-0.3, 1.9) | 0.2 (0.1, 0.3) |
| DLS+ | 7.4 (6.1, 8.9) | 4.4 (3.0, 5.8) | <0.01 | <0.01 | 98.1 (98.0, 98.2) | -1.0 (-1.1, -0.9) | <0.01 | 1 | 3.0 (1.7, 4.3) | 4.0 (2.8, 5.3) | 1.1 (0.9, 1.2) |
| DLS++ | 4.3 (3.3, 5.5) | 1.3 (0.3, 2.3) | <0.01 | <0.01 | 99.0 (98.9, 99.1) | -0.1 (-0.2, -0.0) | <0.01 | 0.998 | 1.2 (0.3, 2.1) | 1.3 (0.4, 2.2) | 0.1 (0.0, 0.2) |
| Metadata | 0.1 (0.0, 0.4) | -3.0 (-3.9, -2.1) | 0.904 | 1 | 100.0 (100.0, 100.0) | 0.9 (0.8, 1.0) | <0.01 | <0.01 | -2.2 (-3.1, -1.4) | -3.1 (-3.9, -2.2) | -0.9 (-0.9, -0.8) |
| Evaluated on the subset with all PPG morphology data available | | | | | | | | | | | |
| Metadata + PPG morphology | 2.2 (1.5, 3.1) | -0.8 (-1.9, 0.2) | <0.01 | 0.977 | 99.5 (99.4, 99.6) | 0.4 (0.3, 0.5) | <0.01 | <0.01 | -0.6 (-1.6, 0.4) | -1.0 (-1.9, 0.0) | -0.4 (-0.5, -0.3) |
| Office-based refit-WHO | 3.0 (2.2, 4.0) | n/a (subset reference) | | | 99.1 (99.0, 99.2) | n/a (subset reference) | | | | | |
| Evaluated on the subset with laboratory data available | | | | | | | | | | | |
| Lab-based refit-WHO | 3.6 (2.7, 4.8) | 0.7 (-0.3, 1.8) | <0.01 | 0.076 | 99.0 (98.9, 99.1) | -0.1 (-0.2, -0.0) | <0.01 | 0.989 | 0.6 (-0.4, 1.6) | 0.7 (-0.3, 1.8) | 0.1 (0.0, 0.2) |
| Office-based refit-WHO | 2.9 (2.1, 3.9) | n/a (subset reference) | | | 99.1 (99.0, 99.2) | n/a (subset reference) | | | | | |
