# Supplementary material for: Predicting cardiovascular disease risk using photoplethysmography and deep learning
Source: PLOS Glob Public Health. 2024 Jun 4;4(6):e0003204. doi: 10.1371/journal.pgph.0003204 (PMC11149850; doi:10.1371/journal.pgph.0003204)
Supplement: S6 Table — (a) We examined the discrimination performance using C-statistic, reclassification improvement using category-free net reclassification improvement (cfNRI), and model calibration using the slope value from the reliability diagram. *In “Feature used” column, “Metadata” includes age, sex, and smoking status. (b) The sensitivity was calculated at the risk threshold matching the specificity of SBP-140, and the specificity was calculated at the risk threshold matching the sensitivity of SBP-140. 95% confidence intervals (CIs) of C-statistic, cfNRI, and slope were obtained from the bootstrapping, and p-values were computed by the permutation test. CIs of sensitivity and specificity were obtained from the Clopper-Pearson exact method, and the p-values were calculated by a permutation test with the prespecified margin of 2.5% and alpha of 0.05. The 95% CIs of NRI were computed by bootstrapping. (DOCX) [file pgph.0003204.s013.docx]

**S6 Table. Model performance comparison of 10-year major adverse cardiovascular events (MACE) risk prediction between DLS versus DLS+ (adding BMI) and DLS++ (adding BMI and SBP).** (a) We examined the discrimination performance using C-statistic, reclassification improvement using category-free net reclassification improvement (cfNRI), and model calibration using the slope value from the reliability diagram. *In “Feature used” column, “Metadata” includes age, sex, and smoking status. (b) The sensitivity was calculated at the risk threshold matching the specificity of SBP-140, and the specificity was calculated at the risk threshold matching the sensitivity of SBP-140. 95% confidence intervals (CIs) of C-statistic, cfNRI, and slope were obtained from the bootstrapping, and p-values were computed by the permutation test. CIs of sensitivity and specificity were obtained from the Clopper-Pearson exact method, and the p-values were calculated by a permutation test with the prespecified margin of 2.5% and alpha of 0.05. The 95% CIs of NRI were computed by bootstrapping.

(A)

| **Model** | **C-statistic (%)** | **Delta in C-statistic (%)** | **P-value for non-inferiority of C-statistic** | **P-value for superiority of C-statistic** | **cfNRI (%)** | **cfNRI (event) (%)** | **cfNRI (non-event) (%)** | **Calibration slope** | **Features used*** |
| --- | --- | --- | --- | --- | --- | --- | --- | --- | --- |
| Office-based refit-WHO | 70.9 (69.7, 72.2) | n/a (reference) | | | | | | 0.979 (0.915, 1.038) | Metadata + BMI + SBP |
| DLS | 71.1 (69.9, 72.4) | 0.2 (-0.4, 0.8) | <0.01 | 0.292 | 0.1 (-0.0, 0.1) | 0.1 (-0.0, 0.2) | 0.0 (0.0, 0.0) | 0.981 (0.919, 1.045) | Metadata + PPG |
| DLS+ | 71.3 (70.2, 72.7) | 0.5 (-0.1, 1.0) | <0.01 | 0.073 | 0.3 (0.2, 0.4) | 0.4 (0.3, 0.5) | 0.1 (0.1, 0.1) | 1.079 (1.001, 1.148) | Metadata + BMI + PPG |
| DLS++ | 71.9 (70.8, 73.2) | 1.0 (0.6, 1.4) | <0.01 | <0.01 | 0.2 (0.1, 0.2) | 0.2 (0.1, 0.2) | -0.0 (-0.0, -0.0) | 0.952 (0.89, 1.01) | Metadata + BMI + SBP + PPG |

(B)

|  | **Sensitivity@specificity of 63.7%** | | | | | | | **Specificity@sensitivity of 55.2%** | | | | | | |
| --- | --- | --- | --- | --- | --- | --- | --- | --- | --- | --- | --- | --- | --- | --- |
| **Model** | **Mean (%)** | **Delta (%)** | **Non-inferiority**  **p-value** | **Superiority p-value** | **NRI (%)** | **NRI (event) (%)** | **NRI (non-event) (%)** | **Mean (%)** | **Delta (%)** | **Non-inferiority**  **p-value** | **Superiority p-value** | **NRI (%)** | **NRI (event) (%)** | **NRI (non-event) (%)** |
| Office-based refit-WHO | 67.7 (65.2, 70.1) | reference | | | | | | 73.1 (72.7, 73.5) | reference | | | | | |
|  | **Sensitivity@specificity of 63.7%** | | | | | | | **Specificity@sensitivity of 55.2%** | | | | | | |
| **Model** | **Mean (%)** | **Delta (%)** | **Non-inferiority**  **p-value** | **Superiority p-value** | **NRI (%)** | **NRI (event) (%)** | **NRI (non-event) (%)** | **Mean (%)** | **Delta (%)** | **Non-inferiority**  **p-value** | **Superiority p-value** | **NRI (%)** | **NRI (event) (%)** | **NRI (non-event) (%)** |
| DLS | 67.9 (65.4, 70.3) | 0.1 (-1.9, 2.0) | 0.012 | 0.654 | -0.3 (-2.0, 1.6) | 1.0 (-0.9, 2.9) | 1.2 (0.9, 1.5) | 74.0 (73.6, 74.4) | 0.9 (-0.7, 2.5) | <0.01 | <0.01 | 1.1 (-0.9, 3.1) | 1.6 (-0.5, 3.4) | 0.4 (0.1, 0.8) |
| DLS+ | 67.9 (65.4, 70.3) | 0.1 (-1.9, 2.2) | <0.01 | 0.5 | 0.0 (-1.9, 2.0) | 0.6 (-1.2, 2.6) | 0.5 (0.2, 0.8) | 74.7 (74.3, 75.0) | 1.4 (-0.2, 2.8) | <0.01 | <0.01 | 2.4 (0.3, 4.3) | 3.5 (1.4, 5.4) | 1.1 (0.8, 1.4) |
| DLS++ | 68.8 (66.3, 71.2) | 1.1 (-0.4, 2.6) | <0.01 | 0.086 | 1.1 (-0.4, 2.5) | 1.3 (-0.1, 2.7) | 0.3 (-0.0, 0.5) | 75.2 (74.8, 75.5) | 2.0 (0.9, 3.1) | <0.01 | <0.01 | 2.6 (1.1, 4.2) | 2.6 (1.1, 4.2) | -0.0 (-0.2, 0.2) |
