# Supplementary material for: Predicting cardiovascular disease risk using photoplethysmography and deep learning
Source: PLOS Glob Public Health. 2024 Jun 4;4(6):e0003204. doi: 10.1371/journal.pgph.0003204 (PMC11149850; doi:10.1371/journal.pgph.0003204)
Supplement: S7 Table — The sensitivity and specificity were calculated at the risk threshold matching SBP-140’s specificity (see Statistical Analysis). 95% confidence intervals (CIs) were obtained from the Clopper-Pearson exact method. (DOCX) [file pgph.0003204.s014.docx]

**S7 Table. Comparison of 10-year major adverse cardiovascular event (MACE) risk prediction performance between different subgroups using DLS versus office-based refit-WHO model.** The sensitivity and specificity were calculated at the risk threshold matching SBP-140’s specificity (see Statistical Analysis). 95% confidence intervals (CIs) were obtained from the Clopper-Pearson exact method.

| Subgroup | Model | C-statistic | Non-inferiority p-value | Superiority p-value | Sensitivity | Specificity | Average predicted risk score | Slope |
| --- | --- | --- | --- | --- | --- | --- | --- | --- |
| Never smoked | Office-based refit-WHO | 72.1 (70.6, 73.9) | - | - | 0.598 (0.562, 0.632) | 0.722 (0.716, 0.727) | 0.023 | 0.73 |
|  | DLS | 71.7 (70.1, 73.3) | 0.11 | 0.815 | 0.558 (0.522, 0.593) | 0.731 (0.726, 0.736) | 0.023 | 0.76 |
| Smoked | Office-based refit-WHO | 68.9 (67.5, 70.4) | - | - | 0.756 (0.725, 0.785) | 0.51 (0.504, 0.517) | 0.035 | 1.01 |
|  | DLS | 69.9 (68.2, 71.3) | <0.01 | 0.029 | 0.786 (0.757, 0.814) | 0.498 (0.491, 0.504) | 0.036 | 0.96 |
| Age<55 | Office-based refit-WHO | 68.7 (66.0, 71.5) | - | - | 0.157 (0.117, 0.205) | 0.936 (0.933, 0.939) | 0.013 | 0.77 |
|  | DLS | 69.1 (66.6, 72.4) | 0.088 | 0.335 | 0.22 (0.174, 0.273) | 0.93 (0.926, 0.933) | 0.014 | 0.86 |
| Age>=55 | Office-based refit-WHO | 65.4 (64.1, 66.8) | - | - | 0.793 (0.77, 0.815) | 0.426 (0.42, 0.431) | 0.038 | 0.95 |
|  | DLS | 65.6 (64.2, 66.8) | <0.01 | 0.335 | 0.775 (0.751, 0.797) | 0.429 (0.424, 0.435) | 0.038 | 0.95 |
| Female | Office-based refit-WHO | 70.8 (68.8, 72.4) | - | - | 0.422 (0.382, 0.463) | 0.82 (0.815, 0.824) | 0.018 | 0.70 |
|  | DLS | 69.5 (68.0, 71.2) | 0.578 | 0.958 | 0.406 (0.366, 0.446) | 0.804 (0.8, 0.809) | 0.019 | 0.78 |
| Male | Office-based refit-WHO | 66.0 (64.5, 67.6) | - | - | 0.836 (0.811, 0.858) | 0.377 (0.37, 0.383) | 0.041 | 0.99 |
|  | DLS | 67.4 (66.0, 68.9) | <0.01 | <0.01 | 0.84 (0.815, 0.862) | 0.396 (0.39, 0.403) | 0.041 | 0.95 |
| HbA1c <=48 | Office-based refit-WHO | 71.0 (69.8, 72.2) | - | - | 0.677 (0.652, 0.701) | 0.636 (0.632, 0.64) | 0.028 | 0.96 |
|  | DLS | 71.2 (69.9, 72.4) | <0.01 | 0.37 | 0.671 (0.646, 0.696) | 0.635 (0.631, 0.639) | 0.028 | 0.95 |
| HbA1c >48 | Office-based refit-WHO | 59.2 (55.7, 62.8) | - | - | 0.701 (0.629, 0.766) | 0.43 (0.407, 0.453) | 0.039 | 0.69 |
|  | DLS | 60.6 (56.6, 64.0) | 0.03 | 0.148 | 0.712 (0.641, 0.776) | 0.447 (0.424, 0.47) | 0.04 | 0.66 |
| No hypertension | Office-based refit-WHO | 70.8 (69.3, 72.4) | - | - | 0.589 (0.556, 0.623) | 0.703 (0.698, 0.707) | 0.024 | 1.03 |
|  | DLS | 71.2 (69.6, 72.9) | <0.01 | 0.134 | 0.613 (0.579, 0.646) | 0.686 (0.681, 0.69) | 0.025 | 1.08 |
| Hypertension | Office-based refit-WHO | 64.4 (62.5, 66.2) | - | - | 0.783 (0.751, 0.812) | 0.42 (0.411, 0.428) | 0.039 | 0.9 |
|  | DLS | 65.3 (63.6, 67.1) | <0.01 | 0.046 | 0.748 (0.715, 0.779) | 0.466 (0.458, 0.475) | 0.037 | 0.84 |
