# Supplementary material for: Predicting cardiovascular disease risk using photoplethysmography and deep learning
Source: PLOS Glob Public Health. 2024 Jun 4;4(6):e0003204. doi: 10.1371/journal.pgph.0003204 (PMC11149850; doi:10.1371/journal.pgph.0003204)
Supplement: S8 Table — Hazard ratios are shown at the median age of the MACE event, which is 63 years in the train split of UKB cohort. Hazard ratios for smokers are for men, and their interaction with sex shows the adjusted risk for women. We included interaction terms between age and other predictors because the HRs for proportional effects on CVD declined with age [2, 3]. (DOCX) [file pgph.0003204.s015.docx]

**S8 Table. Coefficients and hazard ratios from the Cox’s models for 10-year major adverse cardiovascular events (MACE) risk prediction on the UK Biobank (UKB) cohort using DLS, DLS+ and DLS++.** Hazard ratios are shown at the median age of the MACE event, which is 63 years in the train split of UKB cohort. Hazard ratios for smokers are for men, and their interaction with sex shows the adjusted risk for women. We included interaction terms between age and other predictors because the HRs for proportional effects on CVD declined with age [[2,3]](https://paperpile.com/c/hCP1h7/6Jgx+r1iT).

|  | **DLS** | | | | **DLS+** | | | | **DLS++** | | | | **Office-based refit-WHO** | | |
| --- | --- | --- | --- | --- | --- | --- | --- | --- | --- | --- | --- | --- | --- | --- | --- |
| **Predictor** | **Main effect** | **Age interaction term** | **Hazard Ratio** | **PPG feature ranges (mean, SD)** | **Main effect** | **Age interaction term** | **Hazard Ratio** | **PPG feature ranges (mean, SD)** | **Main effect** | **Age interaction term** | **Hazard Ratio** | **PPG feature ranges (mean, SD)** | **Main effect** | **Age interaction term** | **Hazard Ratio** |
| Male Smoker | 0.581 (p=0.094) | -0.0462 (p=0.407) | 1.337 (1.257, 1.422) | - | 0.652 (p=0.061) | -0.0608 (p=0.276) | 1.309 (1.239, 1.382) | - | 0.624 (p=0.075) | -0.0548 (p=0.329) | 1.322 (1.254, 1.395) | - | 0.334 (p=0.061) | -0.0069 (p=0.809) | 1.337 (1.306, 1.37) |
| Female smoker | -0.02309 (p=0.788) | - | 0.977 (0.919, 1.039) | - | -0.02905 (p=0.736) |  | 0.971 (0.92, 1.026) | - | -0.0387 (p=0.653) | - | 0.962 (0.912, 1.015) | - | 0.02069 (p=0.78) | - | 1.021 (0.997, 1.046) |
| Body mass index | - | - | - | - | 0.029 (p=0.38) | 0.0017 (p=0.749) | 1.041 (0.996, 1.087) | - | 0.004 (p=0.896) | 0.0047 (p=0.402) | 1.034 (0.994, 1.076) | - | 0.007 (p=0.649) | 0.0048 (p=0.059) | 1.038 (1.02, 1.056) |
| Systolic blood pressure | - | - | - | - | - | - | - | - | 0.022 (p=0.014) | -0.0025 (p=0.079) | 1.006 (0.982, 1.031) | - | 0.007 (p=0.037) | 0.0002 (p=0.682) | 1.009 (1.0, 1.018) |
| PPG features | | | | | | | | | | | | | - | | |
| PPG-1 | -0.111 (p=0.0) | - | 0.895 (0.879, 0.912) | 0.245 (3.17) | -0.113 (p=0.0) | - | 0.893 (0.874, 0.913) | 0.184 (2.725) | -0.094 (p=0.0) | - | 0.91 (0.893, 0.928) | 0.253 (3.125) |  |  |  |
| PPG-2 | 0.059 (p=0.002) | - | 1.061 (1.021, 1.101) | -0.047 (1.195) | 0.073 (p=0.001) | - | 1.076 (1.031, 1.123) | -0.014 (0.978) | 0.011 (p=0.638) | - | 1.011 (0.966, 1.058) | -0.004 (1.092) |  |  |  |
| PPG-3 | 0.002 (p=0.915) | - | 1.002 (0.965, 1.041) | -0.041 (1.071) | 0.014 (p=0.574) | - | 1.014 (0.966, 1.064) | -0.01 (0.881) | 0.052 (p=0.035) | - | 1.053 (1.004, 1.105) | -0.004 (0.947) |  |  |  |
| PPG-4 | 0.032 (p=0.128) | - | 1.033 (0.991, 1.077) | 0.022 (1.016) | 0.133 (p=0.0) | - | 1.142 (1.082, 1.205) | -0.005 (0.791) | -0.001 (p=0.956) | - | 0.999 (0.953, 1.047) | -0.034 (0.915) |  |  |  |
| PPG-5 | -0.055 (p=0.025) | - | 0.947 (0.902, 0.993) | -0.06 (0.894) | 0.019 (p=0.516) | - | 1.019 (0.962, 1.079) | -0.026 (0.697) | -0.014 (p=0.577) | - | 0.986 (0.937, 1.037) | -0.06 (0.875) |  |  |  |
| PPG-Heart Rate | 0.006 (p=0.001) | - | 1.006 (1.002, 1.009) | - | 0.003 (p=0.124) | - | 1.003 (0.999, 1.006) | - | 0.002 (p=0.245) | - | 1.002 (0.998, 1.006) | - |  |  |  |
