# Supplementary material for: Predicting cardiovascular disease risk using photoplethysmography and deep learning
Source: PLOS Glob Public Health. 2024 Jun 4;4(6):e0003204. doi: 10.1371/journal.pgph.0003204 (PMC11149850; doi:10.1371/journal.pgph.0003204)
Supplement: S9 Table — (DOCX) [file pgph.0003204.s016.docx]

**S9 Table. The list of proxy tasks used for multitask learning.**

| **Variable** | **Type of task** |
| --- | --- |
| Sex | Classification |
| Chronological age | Regression |
| Body mass index (thresholded at 33 kg/m^2^) | Classification |
| Hypertension status | Classification |
| HbA1c (thresholded at 48 mmol/mol / 6.5%) | Classification |
| Total cholesterol (thresholded at 7.16 mmol/L) | Classification |
| Systolic blood pressure (thresholded at 160 mmHg) | Classification |
| Previous MACE event | Classification |
| PPG dicrotic notch | Classification |
