# Supplementary material for: Predicting cardiovascular disease risk using photoplethysmography and deep learning
Source: PLOS Glob Public Health. 2024 Jun 4;4(6):e0003204. doi: 10.1371/journal.pgph.0003204 (PMC11149850; doi:10.1371/journal.pgph.0003204)
Supplement: S10 Table — (a) We examined the ability of discrimination using C-statistic, reclassification improvement using category-free net reclassification improvement (cfNRI), and model calibration using the slope value from the reliability diagram. *In “Feature used” column, “Metadata” includes age, sex, and smoking status. (b) The sensitivity was calculated at the risk threshold matching specificity of the SBP-140 baseline at 63.7%, and the specificity was calculated based on the risk threshold matching sensitivity of the SBP-140 baseline at 55.2%. 95% confidence intervals (CIs) of C-statistic, cfNRI, and slope were obtained from the bootstrapping, and the p-values were computed by the permutation test. CIs of sensitivity and specificity were obtained from the Clopper-Pearson exact method, and the p-values were calculated by the permutation test with the prespecified margin of 2.5% and alpha of 0.05. The 95% CIs of NRI were computed by bootstrapping. (DOCX) [file pgph.0003204.s017.docx]

**S10 Table. Model performance comparison of 10-year major adverse cardiovascular event (MACE) risk prediction between the office-based reference and DLS, and models without smoking status.** (a) We examined the ability of discrimination using C-statistic, reclassification improvement using category-free net reclassification improvement (cfNRI), and model calibration using the slope value from the reliability diagram. *In “Feature used” column, “Metadata” includes age, sex, and smoking status. (b) The sensitivity was calculated at the risk threshold matching specificity of the SBP-140 baseline at 63.7%, and the specificity was calculated based on the risk threshold matching sensitivity of the SBP-140 baseline at 55.2%. 95% confidence intervals (CIs) of C-statistic, cfNRI, and slope were obtained from the bootstrapping, and the p-values were computed by the permutation test. CIs of sensitivity and specificity were obtained from the Clopper-Pearson exact method, and the p-values were calculated by the permutation test with the prespecified margin of 2.5% and alpha of 0.05. The 95% CIs of NRI were computed by bootstrapping.

(A)

| **Model** | **C-statistic (%)** | **Delta (%)** | **P-value for non-inferiority of C-statistic** | **P-value for superiority of C-statistic** | **cfNRI (%)** | **cfNRI (event) (%)** | **cfNRI (non-event) (%)** | **Calibration slope** | **Features used**** |
| --- | --- | --- | --- | --- | --- | --- | --- | --- | --- |
| Office-based refit-WHO | 70.9 (69.7, 72.2) | n/a (reference) | | | | | | 0.979 (0.915, 1.038) | Metadata + BMI + SBP |
| DLS | 71.1 (69.9, 72.4) | 0.2 (-0.4, 0.8) | 0.001 | 0.292 | 0.1 (-0.0, 0.1) | 0.1 (-0.0, 0.2) | 0.0 (0.0, 0.0) | 0.981 (0.919, 1.045) | Metadata + PPG |
| Smoking status-only | 53.9 (52.6, 55.1) | -17.1 (-18.4, -15.5) | 1 | 1 | -1.5 (-1.6, -1.4) | -1.1 (-1.2, -1.0) | 0.4 (0.4, 0.4) | 1.506 (1.153, 2.204) | Smoking |
| Office without smoking status | 70.8 (69.6, 72.1) | -0.1 (-0.3, 0.2) | 0.001 | 0.667 | -0.0 (-0.1, 0.0) | -0.0 (-0.1, 0.0) | -0.0 (-0.0, -0.0) | 0.982 (0.916, 1.051) | Age, sex, BMI, SBP |
| DLS without smoking status | 71.1 (69.9, 72.4) | 0.2 (-0.4, 0.9) | 0.001 | 0.261 | 0.1 (-0.0, 0.2) | 0.1 (-0.0, 0.2) | 0.0 (0.0, 0.0) | 0.968 (0.901, 1.032) | DLS, age, sex |

(B)

|  | **Sensitivity@specificity of 63.7%** | | | | | | | **Specificity@sensitivity of 55.2%** | | | | | | |
| --- | --- | --- | --- | --- | --- | --- | --- | --- | --- | --- | --- | --- | --- | --- |
| Model | Mean (%) | Delta (%) | Non-inferiority  p-value | Superiority p-value | NRI (%) | NRI (event) (%) | NRI (non-event) (%) | Mean (%) | Delta (%) | Non-inferiority  p-value | Superiority p-value | NRI (%) | NRI (event) (%) | NRI (non-event) (%) |
| Office-based refit-WHO | 67.7 (65.2, 70.1) | reference | | | | | | 73.1 (72.7, 73.5) | reference | | | | | |
| DLS | 67.9 (65.4, 70.3) | 0.1 (-1.9, 2.0) | 0.012 | 0.654 | -0.3 (-2.0, 1.6) | 1.0 (-0.9, 2.9) | 1.2 (0.9, 1.5) | 74.0 (73.6, 74.4) | 0.9 (-0.7, 2.5) | <0.01 | <0.01 | 1.1 (-0.9, 3.1) | 1.6 (-0.5, 3.4) | 0.4 (0.1, 0.8) |
| Office without smoking status | 67.6 (65.2, 70.0) | -0.1 (-1.4, 1.1) | <0.01 | 0.5 | 0.3 (-1.1, 1.7) | 1.9 (0.6, 3.3) | 1.7 (1.4, 1.9) | 74.0 (73.7, 74.4) | 0.7 (-0.5, 1.9) | <0.01 | <0.01 | 1.6 (-0.0, 3.1) | 3.1 (1.5, 4.7) | 1.5 (1.3, 1.8) |
| DLS without smoking status | 68.8 (66.3, 71.2) | 1.1 (-0.9, 3.2) | <0.01 | 0.244 | 0.8 (-1.2, 2.8) | 2.0 (-0.1, 3.9) | 1.1 (0.8, 1.4) | 74.2 (73.8, 74.5) | 1.0 (-0.4, 2.5) | <0.01 | <0.01 | 1.2 (-0.7, 3.1) | 1.6 (-0.4, 3.4) | 0.4 (0.0, 0.7) |
