# Supplementary material for: Predicting cardiovascular disease risk using photoplethysmography and deep learning
Source: PLOS Glob Public Health. 2024 Jun 4;4(6):e0003204. doi: 10.1371/journal.pgph.0003204 (PMC11149850; doi:10.1371/journal.pgph.0003204)
Supplement: S1 Data — (DOCX) [file pgph.0003204.s018.docx]

# Supporting references

1. [Ueda P, Woodward M, Lu Y, Hajifathalian K, Al-Wotayan R, Aguilar-Salinas CA, et al. Laboratory-based and office-based risk scores and charts to predict 10-year risk of cardiovascular disease in 182 countries: a pooled analysis of prospective cohorts and health surveys. Lancet Diabetes Endocrinol. 2017;5: 196–213.](http://paperpile.com/b/hCP1h7/Ctml)

2. [Singh GM, Danaei G, Farzadfar F, Stevens GA, Woodward M, Wormser D, et al. The age-specific quantitative effects of metabolic risk factors on cardiovascular diseases and diabetes: a pooled analysis. PLoS One. 2013;8: e65174.](http://paperpile.com/b/hCP1h7/6Jgx)

3. [Hajifathalian K, Ueda P, Lu Y, Woodward M, Ahmadvand A, Aguilar-Salinas CA, et al. A novel risk score to predict cardiovascular disease risk in national populations (Globorisk): a pooled analysis of prospective cohorts and health examination surveys. Lancet Diabetes Endocrinol. 2015;3: 339–355.](http://paperpile.com/b/hCP1h7/r1iT)

4. [Shin H, Noh G, Choi B-M. Photoplethysmogram based vascular aging assessment using the deep convolutional neural network. Sci Rep. 2022;12: 11377.](http://paperpile.com/b/hCP1h7/XoG0)

5. [Pilt K, Meigas K, Ferenets R, Temitski K, Viigimaa M. Photoplethysmographic signal waveform index for detection of increased arterial stiffness. Physiol Meas. 2014;35: 2027–2036.](http://paperpile.com/b/hCP1h7/y4VR)

6. [Allen J. Photoplethysmography and its application in clinical physiological measurement. Physiol Meas. 2007;28: R1–39.](http://paperpile.com/b/hCP1h7/dHyS)

7. [Loh P-R, Tucker G, Bulik-Sullivan BK, Vilhjálmsson BJ, Finucane HK, Salem RM, et al. Efficient Bayesian mixed-model analysis increases association power in large cohorts. Nat Genet. 2015;47: 284–290.](http://paperpile.com/b/hCP1h7/RONs)

8. [Weissbrod O, Hormozdiari F, Benner C, Cui R, Ulirsch J, Gazal S, et al. Functionally informed fine-mapping and polygenic localization of complex trait heritability. Nat Genet. 2020;52: 1355–1363.](http://paperpile.com/b/hCP1h7/teCA)

9. [Hippisley-Cox J, Coupland C, Brindle P. Development and validation of QRISK3 risk prediction algorithms to estimate future risk of cardiovascular disease: prospective cohort study. BMJ. 2017;357: j2099.](http://paperpile.com/b/hCP1h7/pP1M)

10. [Lloyd-Jones DM, Huffman MD, Karmali KN, Sanghavi DM, Wright JS, Pelser C, et al. Estimating Longitudinal Risks and Benefits From Cardiovascular Preventive Therapies Among Medicare Patients: The Million Hearts Longitudinal ASCVD Risk Assessment Tool: A Special Report From the American Heart Association and American College of Cardiology. Circulation. 2017;135: e793–e813.](http://paperpile.com/b/hCP1h7/pHwL)

11. [Loshchilov I, Hutter F. Decoupled weight decay regularization. arXiv preprint arXiv:1711 05101.](http://paperpile.com/b/hCP1h7/U1gE)
