# Supplementary material for: Predicting cardiovascular disease risk using photoplethysmography and deep learning
Source: PLOS Glob Public Health. 2024 Jun 4;4(6):e0003204. doi: 10.1371/journal.pgph.0003204 (PMC11149850; doi:10.1371/journal.pgph.0003204)
Supplement: Attachment — Submitted filename: ppg_rebuttal.docx [file pgph.0003204.s019.docx]

Author Response for “Predicting Cardiovascular Disease Risk using Photoplethysmography and Deep Learning”

Dear Editor and Reviewer,

Thank you for the review of our manuscript entitled “Predicting cardiovascular disease risk using photoplethysmography and deep learning”. We have fully considered your comments and revised the manuscript accordingly. Our detailed point-to-point responses to your comments are listed below, and the updated manuscript is attached.

Regards,

Wei-Hung Weng on behalf of the authors

**Comment: In the introduction section results of the study are also provided in Figure 1.**

Response: Our initial thought was to demonstrate all main results earlier in the manuscript to make our claims clear and easy to capture. However, it may also result in some confusion. Thus we are moving the Kaplan-Meier plots (Fig 1b) and calibration plot (Fig 1c) to Fig 3 and Fig 4, and mention them in the corresponding result sections.

**Comment: The data is from UK biobank and hence how the results can be generalized to lower resource settings?**

Response:

We acknowledge this is an aspect (the link between motivation and the scientific results) of our study that can be clarified and have edited the discussion to make sure this is clear.

This study required longitudinal data collected from PPG sensors and also long-term outcomes data such as the presence of major adverse cardiac events within 10 years. There currently are no datasets containing PPG data and long term outcomes in lower resource settings. This may be because it is resource-intensive to collect such data.

In order for this data to be collected, there needs to be an assessment of the feasibility and value of the approach, and development of tooling in order to make it possible. Our work aims to address this in two key ways.

1. We believe our work with the UKB data provides a feasibility signal by proving there is a cardiovascular disease risk signal in PPG. This will make it easier to justify inclusion of PPG data collection in studies that may track long term outcomes.
2. To make collection of the necessary longitudinal data easier, we have now open sourced a mobile PPG data collection app (<https://github.com/google-research/CVD-paper-mobile-camera-example>) which facilitates the inclusion of mobile PPG data collection in any studies going forward. This lowers the technical and logistical barriers to adding PPG data collection to any existing longitudinal studies collecting long term outcomes data in lower resource settings by making it possible to collect PPG data using just a smartphone. We have added mention of this into the publication.

By addressing these two key points we believe that our publication represents a meaningful step towards collecting the key data that does not exist today for generalization to lower resource settings. We have edited the discussion to clarify this.

**Comment: Cox proportional hazard was used to predict the ten-year risk of MACE among individuals free of CVD at baseline. Whether proportionality assumptions are satisfied? The predictors considered were using age, sex, smoking status and the results of analysis of PPG signals using deep learning.**

Response: Of five PPG features, one feature (PPG-2) yielded a p-value <0.05 for the non-proportional test. When correcting for multiple testing (alpha of 0.01), this is not significant. The slope and residuals of PPG-2 are near zero via the Schoenfield residual plot, indicating a low effect size. The literature also supports that proportional hazard assumption may be violated given a large enough sample size [1].

To investigate further, PPG-2 indeed demonstrates a significant main effect in the model (S3 Table), and it also explains a reasonable percentage of the variance (see the response in the comment below about PCA).

As a further sensitivity analysis, we found that removing or binning PPG-2 can resolve the proportional assumption issue, and even increase the test subset performance (C-statistic below). Given the statistical analysis above, we have added these results to the caption of Table 2.

- Original DLS: 71.1 (69.9, 72.4)
- Remove PPG-2: 72.0 (70.8, 73.2)
- Binning PPG-2: 71.9 (70.8, 73.2)

**Comment: The authors mentioned that the dataset was divided in to three train, tune and test based on latitude and longitude. But how this division was made based on latitude and longitude was not explained.**

Response: We have added more details of the geographical site information, including region, latitude and longitude in the S10 Table (with the new visualization in S5 Fig and below), and highlight the corresponding regions in Table 1. We have also mentioned the motivation of using the geographical splitting strategy in the main text Data source and cohort section: “We then stratified UKB participants who had PPG waveforms recorded into three subsets: train (n=105,319), tune (n=46,868), and test (n=57,702) subsets based on geographic information on the site of data collection, i.e., latitude and longitude. This strategy aligns with TRIPOD guidelines [24] on external validation (specifically validation on a different geographic region) by allowing for non-random variation between data splits such as differences in data acquisition or environment.”

**Comment: In Table 1 what is the meaning of “On hypertension medication, median [IQR]”. It is a count and not median. Similarly, for “On statin, median [IQR]”.**

Response: Thank you for catching these. We have corrected them accordingly in the updated manuscript.

**Comment: On an average how many follow-up visits were there for a participant?**

Response: There are 0.169, 0.061, 0.037 follow-up visits on average in the train, tune, and test cohort subsets, respectively. However, we would like to clarify that we only use the first visit since the goal of our model is to provide a risk prediction using a single data point rather than sequential information. There are also potential differences in participants who attend follow-up visits (as a trivial example, they must not have had a fatal cardiac event after the first visit).

**Comment: What is the percentage of censoring in training, tune and test data used for the data analysis?**

Response: The percentage of right-censoring is 97.1% (95173 / 97970), 96.8% (42138 / 43539), and 97.3% (53405 / 54865) in the train, tune, and test cohort subsets, respectively. We have added this information into Table 1.

**Comment: What was the criteria for selecting five Principal components?**

Response: We decided to use five principal components before running all study experiments and analyses to avoid post-hoc selection. We chose five components based on pilot experiments where they usually explained more than 60% of the variance, and led to all features having reasonably low p-values in multivariable analysis. In the next response, we compute the variance that can be explained by the five components in our models.

**Comment: What is the total percentage of variance explained by these five components?**

Response: The five principal components (PC) of PPG representations in DLS, DLS+, and DLS++ explain a cumulative 67.8%, 74.9%, and 69.4% of the variance, respectively. More details per PC:

- Top-5 components in DLS explain 46.4%, 7.0%, 5.55, 4.9%, 3.9% of the variance.
- Top-5 components in DLS+ explain 53.5%, 7.2%, 5.8%, 4.7%, 3.6% of the variance.
- Top-5 components in DLS++ explain 49.8%, 6.4%, 4.8%, 4.4%, 4.1% of the variance.

**Comment: The DLS yielded C-statistic value is only 71.1% though is it significant, the C value is very low.**

Response: We acknowledge that the C-statistic of the DLS is not as high as may be expected for diagnostic models. However, this is not unusual for prognostic models focusing on long-term outcomes. For example, the currently used valid cardiovascular risk prediction scores all have AUC (binary analog of C-statistic) in the range of 70% to 80% [2]. In our study, we found that office-based and lab-based scores using WHO or Globorisk features also yielded the C-statistic <72%. Globorisk (WHO) refitted models yielded 70.9% (office-based) and 71.6% (lab-based) in our evaluation cohort (Table 2). The Full model we examined using most risk factors in QRISK and/or ASCVD yielded a C-statistic of 73.5% (in Supporting results). From the literature, Framingham cardiovascular right algorithm yielded a slightly higher C-statistic of 76.3% for men and 79.3% for women [3], yet it requires relatively costly laboratory measurements for total cholesterol and HDL, which are beyond the scope of the low-medical resource setup. Another machine learning based risk model, AutoPrognosis, yielded 77.4% AUC on UKB. Yet this model includes 473 features (including more expensive lab features), and the evaluation cohort is different from ours [4]. The Globorisk score, which is designed for low-medical resource setting, yielded a C-statistic of 70% in the literature, and can discriminate well in internal and external validations [5]. Even though high AUC (C-statistic) is required for a diagnostic tool, a relatively modest AUC (C-statistic) doesn’t preclude a score from being used as a screening or triaging tool, which is the main purpose of our proposed model. Thus, we believe that our model still can be considered as a good proof-of-concept screening and triaging tool for monitoring cardiovascular risk especially for the medical resource-limited regions.

**Comment: Figures are of poor clarity.**

Response: We have confirmed with the editor, and this is an artifact of the PDF generation, and the original figures are of high enough quality.

## Reference

[1] Stensrud MJ, Hernán MA. Why test for proportional hazards?. JAMA. 2020 Apr 14;323(14):1401-2.

[2] Lloyd-Jones DM. Cardiovascular risk prediction: basic concepts, current status, and future directions. Circulation. 2010 Apr 20;121(15):1768-77.

[3] D’Agostino Sr RB, Vasan RS, Pencina MJ, Wolf PA, Cobain M, Massaro JM, Kannel WB. General cardiovascular risk profile for use in primary care: the Framingham Heart Study. Circulation. 2008 Feb 12;117(6):743-53.

[4] Alaa AM, Bolton T, Di Angelantonio E, Rudd JH, Van der Schaar M. Cardiovascular disease risk prediction using automated machine learning: A prospective study of 423,604 UK Biobank participants. PloS ONE. 2019 May 15;14(5):e0213653.

[5] Hajifathalian K, Ueda P, Lu Y, Woodward M, Ahmadvand A, Aguilar-Salinas CA, Azizi F, Cifkova R, Di Cesare M, Eriksen L, Farzadfar F. A novel risk score to predict cardiovascular disease risk in national populations (Globorisk): a pooled analysis of prospective cohorts and health examination surveys. The Lancet Diabetes & endocrinology. 2015 May 1;3(5):339-55.
