# Supplementary material for: Predicting cardiovascular disease risk using photoplethysmography and deep learning
Source: PLOS Glob Public Health. 2024 Jun 4;4(6):e0003204. doi: 10.1371/journal.pgph.0003204 (PMC11149850; doi:10.1371/journal.pgph.0003204)
Supplement: Attachment — Submitted filename: ppg_response_r2.docx [file pgph.0003204.s020.docx]

Author Responses for “Predicting Cardiovascular Disease Risk using Photoplethysmography and Deep Learning”

Dear Editor and Reviewers,

Thank you for the review of our manuscript entitled *“Predicting cardiovascular disease risk using photoplethysmography and deep learning”*. We have fully considered your comments and revised the manuscript accordingly. Our detailed point-to-point responses to your comments are listed below, and the updated manuscript is attached.

Regards,

Wei-Hung Weng, Sebastien Baur, Mayank Daswani on behalf of the authors

# Reviewer #2

## Comment: A lot of information related to model development is missing from the paper. Describe in detail which patterns of PPG and which input format one-dimensional ResNet18 analyzed.

Response:

- We acknowledge that more details of the PPG input format can be added into the manuscript for reproducibility. The PPG waveform is taken from the UKB Data-Field 4205 (<https://biobank.ndph.ox.ac.uk/ukb/field.cgi?id=4205>), which were collected by a specific PPG device, PulseTrace PCA2 (Manual: <https://docplayer.es/23504573-Pulsetrace-pca2-operating-manual-english-manual-operativo-espanol.html>).
- We have added the description of the raw data and the model input after the edited sentence *“For DLS, we first trained a deep learning-based feature extractor to learn PPG representations from UKB PPG summarized waveform signals, using a one-dimensional ResNet18 (He et al. 2016; Dall'Olio et al. 2020; Shin et al. 2022) as the neural network architecture”,* in the Model development section: *“Specifically, the UKB PPG waveform signals (UKB Data-Field 4205) was collected by the PPG device, PulseTrace PCA2 (CareFusion, USA), and the device averaged a minimum of six pulse waveforms with a pulse interval close to the average pulse interval, and output a single summarized waveform stretched to 100 temporal units irrespective of heart rate (for example, see* [*https://biobank.ndph.ox.ac.uk/ukb/refer.cgi?id=100181*](https://biobank.ndph.ox.ac.uk/ukb/refer.cgi?id=100181)*). Since the UKB normalized the PPG data into 100-length time-series irrespective of the heart rate, the time interval between successive values can vary across participants due to variations in heart rate. Thus, we resampled the x-axis of the raw data so that the interval between two timesteps is uniform (18.2 milliseconds per step), resulting in variable-length time-series per participant. We then padded all samples to the same length with zeros. We also applied the Brownian-tape speed data augmentation (details in the Supporting text, the “Details of model training” section), which is specific to time-series data.”*

## Comment: PPG is a time series signal. In general, performance improves by applying preprocessing related to signal processing or models related to time series analysis. Please describe whether any processing related to this was done.

Response: We have now added sentences in the Model development section to mention the time-series specific data processing that we have done: *“Since the UKB normalized the PPG data into 100-length time-series irrespective of the heart rate, the time interval between successive values can vary across participants due to variations in heart rate. Thus, we resampled the x-axis of the raw data so that the interval between two timesteps is uniform (18.2 milliseconds per step), resulting in variable-length time-series per participant. We then padded all samples to the same length with zeros. We also applied the Brownian-tape speed data augmentation (details in the Supporting text, the “Details of model training” section), which is specific to time-series data.*

## Comment: Results must be shown regarding the interpretability of the deep learning model.

Response: Regarding interpreting the developed models, S4 Table has shown the coefficients and hazards ratios of the DLS PPG features in the Cox model. Also, the Discussion section currently mentions *“We computed the Pearson correlation coefficient between DLS features and engineered PPG morphological features, and found some correlations exceeding 0.3.”*. To make it clearer, we further added *“For example, we found that the correlation between PPG-1 and peak-to-peak time, peak position, shoulder position is 0.359, -0.474, -0.527, respectively, the correlation between PPG-2 and peak-to-peak time, and dicrotic notch position is 0.369, 0.343, respectively, and the correlation between PPG-4 and shoulder position is 0.361. Even though the correlations may provide some interpretability, further investigation is still required for interpreting the deep learning-based features.”*

## Comment: Signal measurement equipment such as PPG is subject to enormous bias depending on the equipment manufacturers. Describe UKB's PPG and other sets' equipment accurately and discuss bias.

Response:

- We agree that understanding differences between equipment used to collect data is important. However, because PulseTrace PCA2 is the UKB-determined device, we are unable to assess differences between manufacturers. That said, because we split the data geographically, and each site uses its own device (of the same model), our results are demonstrating generalization across any device-specific calibration. We also included this new sentence: *“Future work may be needed to understand how to mitigate any differences in PPG features based on PPG device or manufacturer.”*
- In the Discussion section, we have modified the following sentence as a limitation of our study: “*The device used for PPG acquisition across the UKB is a specific clinical pulse oximeter (PulseTrace PCA2), thus our results provide direct evidence that waveforms from this pulse oximeter may be a reliable CVD screening tool. Studies have found that the heart rate and rhythm extracted from smartphone PPG were comparable with clinical grade devices such as ECG (Li et al. 2019; Plews et al. 2017; De Ridder et al. 2018), but additional work is needed to know if our model would transfer to smartphone-collected PPGs.”*
- In the Model Development section, we now include a description of the UKB PPG signal (see comments 1 and 2), which is measured via pulse oximetry and is processed in a certain way. In the Discussion section, we further added a mention: “*Since the PPG waveform signals in the UKB have only been collected by a single device and specific protocol (details in Model development section), further work may be necessary to understand if these data are biased in some way (e.g., less noisy) relative to less structured data collection protocols.*”

# Reviewer #3

## Comment: There is a major concern on the scientific basis underlying the results. It is known that the quality and morphology of PPG signals are highly sensitive to noises, and are dependent on many factors including wavelength, sensor attachment pressure, measurement site, and other physiological conditions, as well as the pre-processing method. The instability of PPG waveform is a major limitation in clinical application. In comparison, ECG morphology is more reliable. The proposed DLS contain important risk factors. The efficacy of PPG need to be evaluated independently.

Response:

- We agree that free-living subjects’ PPG signals from other devices may contain additional noise relative to the structured collection in the UKB, and further evaluation is needed. We have added a mention: *“Since the PPG waveform signals in the UKB have only been collected by a single device and specific protocol (details in Model development section), further work may be necessary to understand if these data are biased in some way (e.g., less noisy) relative to less structured data collection protocols.”* in the Discussion section.
- Regarding noise and measurement methodology, we have now added the details of UKB PPG collection protocol and our processing information in the Model development section: *“Specifically, the UKB PPG waveform signals (UKB Data-Field 4205) was collected by the PPG device, PulseTrace PCA2 (CareFusion, USA), and the device averaged a minimum of six pulse waveforms with a pulse interval close to the average pulse interval, and output a single summarized waveform stretched to 100 temporal units irrespective of heart rate (for example, see* [*https://biobank.ndph.ox.ac.uk/ukb/refer.cgi?id=100181*](https://biobank.ndph.ox.ac.uk/ukb/refer.cgi?id=100181)*). Since the UKB normalized the PPG data into 100-length time-series irrespective of the heart rate, the time interval between successive values can vary across participants due to variations in heart rate. Thus, we resampled the x-axis of the raw data so that the interval between two timesteps is uniform (18.2 milliseconds per step), resulting in variable-length time-series per participant. We then padded all samples to the same length with zeros. We also applied the Brownian-tape speed data augmentation (details in the Supporting text, the “Details of model training” section), which is specific to time-series data.”*
- We agree that the ECG morphology provides a reliable signal for cardiovascular pathologies/conditions and is widely used in several forms including 12-lead and Holter. The literature also presents evidence that the PPG can be as useful as ECG for some applications: *“Studies have found that the heart rate and rhythm extracted from smartphone PPG were comparable with clinical grade devices such as ECG [41–43]”.* We couldn’t make a direct comparison between PPG and ECG in our study since in UKB the subset with both PPG and ECG is too small (less than 30 positive MACE cases for evaluation). However, we have edited the Discussion section to describe that we found ECG is comparable with the office-based Globorisk score yet further evidence and data collection are required to make it conclusive (*“We also found preliminary evidence that using a resting electrocardiogram (ECG) yielded a comparable performance to the office-based model on a UKB subset with available resting ECG data.”*).
- On a related note, we also believe that there is a clearer path towards widespread use in low-resource environments for PPG-based risk scores than for ECG-based scores since PPG can be captured by mobile devices more easily and cheaply than ECG given that the cameras are ubiquitous on smartphones whereas ECG requires special conductive sensors.
- On a related note, we have also examined the performance using solely PPG feature (S3 Table). The C-statistic of the PPG-only model is 62.7 (DLS: 71.1, office-based Globorisk: 70.9) (added in the Result section: “*With only five deep learning-based PPG features, the C-statistic is 62.7% (61.3, 63.9).*”). However, we would like to emphasize that the goal of the study is not for PPG to be used alone, but to evaluate the potential to replace the blood pressure measurement (which requires a medical device).

## Comment: Regarding the term "prediction", the time of follow-up is an important concern. The PPG morphology is age-dependent so age can be a confounding factor.

Response:

- We acknowledge that the ten-year horizon is an important factor that makes it hard to further validate our methodology since there are no public datasets that currently exist outside of UKB. One of the key objectives of this study is that we hope to encourage researchers to collect PPG measurements as part of their longitudinal studies, so that this question can be answered more thoroughly.
- We also agree that age can be a confounding factor. To understand this better, we have evaluated the effect of adding and removing age on the C-statistic below. We show that the survival model using PPG + age did better than age alone (see the Table below).

| **Model** | **C-statistic (95% CI)** |
| --- | --- |
| PPG-only | 62.7 (61.3, 63.9) |
| Age-only | 66.1 (64.8, 67.4) |
| **PPG + age** | **66.8 (65.5, 68.0)** |

# Editor

## Comment: Include a detailed review of previous studies using photoplethysmography in cardiovascular risk assessment.

Response: Since there is no existing works on predicting CVD risk using PPG, we added the following in the Introduction section: *“While there are existing works on the BP estimation and evaluating other related CVD risk-factors such as heart rate variability (HRV) or arterial stiffness from PPG (Natarajan et al. 2020; Charlton et al. 2022), we have not found existing literature on predicting CVD risk directly from PPG waveform signals. The closest related works are those who predicted CVD risk via arterial stiffness index (ASI) estimated by PPG (Inoue et al. 2017; Hellqvist et al. 2019; Gunarathne et al. 2008).”*

## Comment: Provide justification for the chosen deep learning model architecture instead of conventional analytical choice. Explain the data division process for training, validation, and testing.

Response:

- Regarding the chosen model architecture, one-dimensional convolutional neural networks (1D-CNNs) such as 1D-ResNet used in the study is known to be an useful modeling technique for time-series related tasks [1, 2, 3]. More advanced neural network architectures may yield higher performance for time-series data yet also require larger computational resources, such as autoregressive models like WaveNet [4], or other Transformer-based models. These models may not be ideal for our motivation of adopting the technique in low-resource settings since the model needs to run on mobile devices. Thus, we believe that lightweight models like 1D-ResNet18 would be a better choice. We have added a sentence to emphasize the reason of choice in the Model development section: “We selected the one-dimensional ResNet18 as the backbone for time-series modeling since one-dimensional convolutional neural networks are known to be a strong model for time-series tasks (Ismail Fawaz et al. 2019), and one-dimensional ResNet18 is lightweight for potential low-resource setting use, such as deploying on mobile devices.”
- The conventional approach may be using patient metadata and hand-crafted UKB PPG morphological features (such as presence and position of dicrotic notch), as we have analyzed in the manuscript (see Metadata + PPG morphology). We have shown that this approach was not robust enough and underperformed the deep learning-based methods (Table 2: C-statistic of 70.0 for metadata + PPG morphology, versus 70.9 for DLS). We also added descriptions of the correlation between deep learning-based PPG features and the hand-crafted morphological features in the Discussion section: *“For example, we found that the correlation between PPG-1 and peak-to-peak time, peak position, shoulder position is 0.359, -0.474, -0.527, respectively, the correlation between PPG-2 and peak-to-peak time, and dicrotic notch position is 0.369, 0.343, respectively, and the correlation between PPG-4 and shoulder position is 0.361. Even though the correlations may provide some interpretability, further investigation is still required for interpreting the deep learning-based features.”*
- The data division process has been clarified in the Material and methods, Data source and cohort section: *“We then stratified UKB participants who had PPG waveforms recorded into three subsets: train (n=105,319), tune (n=46,868), and test (n=57,702) subsets based on geographic information on the site of data collection, i.e., latitude and longitude. This strategy aligns with TRIPOD guidelines [24] on external validation (specifically validation on a different geographic region) by allowing for non-random variation between data splits such as differences in data acquisition or environment.”* The details of splits are also listed in Table 1, S10 Table, and S5 Fig.

## Comment: Show the comparison with existing cardiovascular risk scores with the approach chosen by the authors.

Response: We agree it should have been clearer that we made comparisons between our developed models versus both office-based and lab-based Globorisk (which are the scores people use in the context of low-resource medical settings). We have now added the following in the caption of Fig 1: “*In this study, we compare our developed model (DLS) with the existing office-based and lab-based CVD risk scores that have been developed for low-resource medical settings, including models refitting variables in the WHO and Globorisk scores, and office-based and lab-based Globorisk scores recalibrated on the same study cohort.*”, and adding the following in the caption of Fig 4: “*METADATA: the risk model with age, sex, smoking status), OFFICE: the risk model using shared predictors from the office-based WHO/ISH risk chart and Globorisk score. DLS: the risk model with metadata and deep learning-based PPG features. DLS+: the risk model using all DLS predictors plus BMI. DLS++: the risk model using all DLS predictors plus BMI and systolic blood pressure.*”. We also added “*(details in the Supplementary results)*” while describing “Full” model in the Model development section, to reflect the comparison to a model with features comparable with the QRISK score in the Supplementary results (Section *“Applying additional features helps improve the cardiovascular disease risk prediction”*, Full model). We only provide details in the Supplementary material since QRISK requires many expensive lab measurements which would not be available in low-resource settings.

## Comment: Discussion can benefit from clinical implications given the study’s limitations. Also, please discuss potential future research directions in the conclusion.

Response: Thank you for the suggestion. Regarding the clinical implications, we have edited the Discussion section: “*Additional work will also be needed to know if our model would transfer the smartphone setup, because the smartphone PPG datasets with longitudinal MACE outcomes in LMIC do not exist to our knowledge, so direct evidence of the efficacy of such an approach will need to be evaluated when such data become available.*” Regarding potential future directions, we agree it would be valuable to highlight and have edited the conclusion: “*Our work is an initial step towards accurate and scalable CVD screening in resource-limited areas around the world, and will hopefully inspire the collection of real-world datasets with smartphone-acquired PPG and longitudinal outcomes.*” Additionally, we added the following to reflect our future plan from the technical perspective: “*We have also investigated other larger network architectures such as the dilated CNN and WaveNet yet without marked performance improvement, further investigation on the efficient modeling that can improve the performance yet also being lightweight will be considered.*”

# Reference

[1] Ismail Fawaz H, Forestier G, Weber J, Idoumghar L, Muller PA. Deep learning for time series classification: a review. Data mining and knowledge discovery. 2019 Jul;33(4):917-63.

[2] Wang Z, Yan W, Oates T. Time series classification from scratch with deep neural networks: A strong baseline. In2017 International joint conference on neural networks (IJCNN) 2017 May 14 (pp. 1578-1585). IEEE.

[3] Tang W, Long G, Liu L, Zhou T, Jiang J, Blumenstein M. Rethinking 1d-cnn for time series classification: A stronger baseline. arXiv preprint arXiv:2002.10061. 2020 Feb:1-7.

[4] Oord AV, Dieleman S, Zen H, Simonyan K, Vinyals O, Graves A, Kalchbrenner N, Senior A, Kavukcuoglu K. Wavenet: A generative model for raw audio. arXiv preprint arXiv:1609.03499. 2016 Sep 12.
