# Supplementary material for: Predicting cardiovascular disease risk using photoplethysmography and deep learning
Source: PLOS Glob Public Health. 2024 Jun 4;4(6):e0003204. doi: 10.1371/journal.pgph.0003204 (PMC11149850; doi:10.1371/journal.pgph.0003204)
Supplement: Attachment — Submitted filename: ppg_response_r3.docx [file pgph.0003204.s021.docx]

Author Responses for “Predicting Cardiovascular Disease Risk using Photoplethysmography and Deep Learning”

Dear Editor and Reviewers,

Thank you for the review of our manuscript entitled *“Predicting cardiovascular disease risk using photoplethysmography and deep learning”*. We have considered all your comment and revised the manuscript accordingly. Our detailed response to the reviewer's comment is listed below, and the updated manuscript is attached.

Regards,

Wei-Hung Weng, Sebastien Baur, Mayank Daswani on behalf of the authors

# Reviewer #2

## Comment: This study is expected to have high impact due to the big data analysis conducted by Google. For this reason, a more kind explanation is needed. Although there are coefficients for features in the supplementary material, the model in the presented paper is not explainable. Regardless of the interpretation method, an interpretable explanation of which patterns are most closely related to cardiovascular disease is required along with representative images or sample signals of the analyzed PPG. Leaving the model developed in the paper as a blackbox reduces the value of the research. I suggest that authors present representative PPG figures that many researchers can understand. Additionally, it is necessary to check throughout the paper whether supplementary materials are appropriately mentioned. There seem to be items that are numbered incorrectly or not mentioned.

Response: Thank you for underscoring the importance of, and the suggestion for more additional interpretation results. As noted, the paper currently includes coefficients for features (in S8 Table), and the correlation calculation between DLS PPG features and known UKB PPG features (in Discussion). We are further adding several techniques below, including both representative PPG figures to understand each feature, and accompanying saliency plots in the revised manuscript. Specifically, we have added paragraphs in the end of the result section discussing the findings of interpretability along with a table (Table 4) showing the correlation between DLS PPG features versus the known UKB PPG features (originally few sentences in the discussion section), and a figure (Fig 5) demonstrating the waveforms and saliency maps:

- *To understand the DLS model deeper, we analyzed the DLS-based PPG features via coefficients and hazard ratios (HRs), correlations with known PPG morphological features, the difference of PPG waveforms between high/low values of DLS PPG feature, and saliency map of the PPG features, computed using integrated gradients [1] of the Cox log partial hazard with respect to the input waveform, using the same metadata and linear interpolation from a constant (all zeros) baseline for the PPG. We first examined the association between each model and MACE via the coefficients and hazard ratios (HRs) (S8 Table). We found that in the office-based refit-WHO score, smoking, older age, higher BMI, and higher SBP were associated with ten-year MACE risk. We found that some DLS features were also associated with ten-year MACE risk (p<0.05 for four deep learning PPG features in DLS and DLS+, and for two PPG features in DLS++). Next, we computed the Spearman’s rank correlation coefficient between DLS features and engineered PPG morphological features (Table 4), and visualized the relationship between waveforms and the PPG feature values / predicted risk score change, along with the integrated gradients.*
- ***Table 4. Spearman’s rank correlation coefficients between each PPG feature and annotated PPG features from the UK Biobank.*** *The comments column summarizes observations about these features. Pulse rate is the heart rate during arterial stiffness measurement. Reflection index is a measure of reflection, the ratio between reflected wave peak and the direct wave peak height. Peak-to-peak time is the difference between the peak values of direct and reflected components. Peak position is the time when the peak occurs in the waveform. Notch position is the time when notch occurs in the waveform. Shoulder position is the time of the shoulder-like curve in the waveform, and Stiffness index is calculated by the peak-to-peak time divided into the person's height.*

| DLS PPG Feature | Univariable Cox coefficient for DLS feature | Multivariable Cox coefficient for DLS feature | Correlation of DLS feature with UK Biobank PPG feature | | | | | | | Comments |
| --- | --- | --- | --- | --- | --- | --- | --- | --- | --- | --- |
|  |  |  | Pulse rate  (0.077)* | Reflection index (0.052)* | Peak-to-peak time (-0.232)* | Position of the peak (0.281)* | Position of the notch (-0.009)* | Position of the shoulder (0.281)* | Stiffness index (0.027)* |  |
| PPG-1 | -0.154 | -0.111 | 0.074 | -0.16 | 0.48 | -0.48 | 0.061 | -0.539 | -0.43 | Correlation with peak position, systolic peak width, narrow clearer notch |
| PPG-2 | 0.034 | 0.059 | 0.18 | -0.438 | 0.373 | -0.002 | 0.293 | -0.177 | -0.418 | Correlation with peak-to-peak time |
| PPG-3 | 0.065 | 0.002 | 0.114 | -0.114 | 0.068 | -0.127 | 0.121 | -0.021 | -0.033 | Weakly correlated to known PPG features |
| PPG-4 | 0.010 | 0.032 | 0.147 | -0.121 | -0.095 | 0.273 | 0.28 | 0.354 | 0.066 | Correlation with shoulder and peak position |
| PPG-5 | 0.063 | -0.055 | -0.041 | 0.227 | 0.02 | -0.047 | 0.012 | 0.024 | 0.032 | Correlation with reflection index |

- Univariable Cox coefficients of the UK Biobank PPG feature after normalizing to zero mean and unit variance, to help understand directionality of association with MACE.
- *In Fig 5, we have visualized the PPG waveforms based on the predicted risk score and five DLS PPG feature values, by presenting the average of 100 PPG waveforms sampled nearest to the following quantiles: 10th, 50th, and 90th. We found that with higher PPG-1 value, the systolic peak shifts earlier; notch appears more prominent. A leftward systolic peak shift is consistent with a higher slope and thus less stiff vessels, corroborated by the strong positive correlation with the peak-to-peak time feature and negative correlation with the stiffness index. Together, these findings are consistent with the risk prediction observations above because PPG-1 has the highest absolute Cox coefficient among the five DLS PPG features, and the direction is negative (hence inverse direction with the observations in the predicted score). PPG-2 is also correlated with peak-to-peak time and stiffness index, and the cases with higher PPG-2 values show a lower waveform and notch amplitude, which may be consistent with the negative correlation with reflection index, though other morphological changes along this spectrum are harder to describe precisely. PPG-3 is weakly correlated with cardiac events (it has a small Cox coefficient), and weakly correlated with the available UK Biobank-provided PPG features, we also didn’t see a significant difference between waveforms with the change of PPG-3 value. PPG-4 is correlated with the shoulder position and more weakly, with systolic peak position. The morphological differences at the notch are subtle, though the leftward shift is consistent from 10th to 50th to 90th percentile. PPG-5 appears correlated to changes in the relative notch height, which is consistent with its high correlation with reflection index. The reflection index is in turn a measure of peripheral resistance, and it also tends to be more common in some specific age group (40-54) and obese groups [2], and it also loosely correlates with other risk predictor metric such as pulse wave velocity (PWV) [3]. Interestingly, PPG-5 is the only PPG feature which had a positive univariable association (positive Cox coefficient) with MACE outcomes, but which became a negative association in multivariable analysis. This likely reflects its effect being that of modulating other features. We also found that stratifying by the DLS predicted risk score, the systolic peak shifts later with higher risk prediction and notch appears to be less prominent, which is also related to vascular stiffness. Finally, we found that saliency maps, computed using integrated gradients, (Fig 5 right) highlighted the waveform’s peak, notch and diastolic phase as the areas most responsible for changes in predicted risk.*
- ***Fig 5. DLS PPG features explainability visualizations.*** *The first row sorts PPGs by predicted risk, whereas the next 5 rows sort PPGs based on the 5 PPG feature values. The first column presents the average of 100 PPG waveforms sampled nearest to the following quantiles of the quantity mentioned on the left: 10th (red) 50th (green) and 90th (blue). The next 3 columns present the respective averaged PPGs along with the normalized saliency values based on integrated gradients. We observed that in general salient areas that most influence the predictions seem to be near the top of the systolic peak and the notch, independent of which quantile and feature/prediction the PPG was sampled from. Each PPG feature appears to correspond to different morphological aspects (see Table 4).*

Finally, we have also carefully gone through the revised manuscript, and updated all tables, figures, and supplementary materials mentioned in the correct order based on the latest revision.

# Reference

[1] Sundararajan M, Taly A, Yan Q. Axiomatic attribution for deep networks. InInternational conference on machine learning 2017 Jul 17 (pp. 3319-3328). PMLR.

[2] Tąpolska M, Spałek M, Szybowicz U, Domin R, Owsik K, Sochacka K, Skrypnik D, Bogdański P, Owecki M. Arterial stiffness parameters correlate with estimated cardiovascular risk in humans: a clinical study. International Journal of Environmental Research and Public Health. 2019 Jul;16(14):2547.

[3] Padilla JM, Berjano EJ, Saiz J, Facila L, Diaz P, Merce S. Assessment of relationships between blood pressure, pulse wave velocity and digital volume pulse. In2006 Computers in Cardiology 2006 Sep 17 (pp. 893-896). IEEE.
